# Development and internal validation of an interpretable machine-learning model for identifying comorbid atrial fibrillation in patients with diabetic kidney disease

**DOI:** 10.3389/fcdhc.2026.1785125

**Published:** 2026-05-26

**Authors:** Xiaoran Li, Xueying Wang, Shidong Wang, Xuebing Zhang

**Affiliations:** 1 Department of Endocrinology, Dongzhimen Hospital Affiliated to Beijing University of Chinese Medicine, Beijing, China; 2 Department of Geriatrics, Beijing Electric Power Hospital, Beijing, China

**Keywords:** atrial fibrillation (AF), comorbidity identification, diabetic kidney disease (DKD), machine learning (ML), Shapley additive explanations (SHAP)

## Abstract

**Background:**

Patients with diabetic kidney disease (DKD) are at increased risk of atrial fibrillation (AF), yet tools to support identification of comorbid AF in this high-burden population remain limited. We aimed to develop and internally validate an interpretable machine-learning (ML) model for identifying concomitant AF in patients with DKD using routinely collected clinical data.

**Methods:**

In this retrospective two-center cohort study (January 2021 to October 2025), 787 unique records of patients with DKD were randomly divided into training (70%) and test (30%) sets. AF status was defined as documented atrial fibrillation coexisting with DKD and was ascertained using electrocardiograms, Holter monitoring when available, and ICD-10 diagnostic codes with physician adjudication. Candidate predictors were routine clinical, laboratory, and echocardiographic variables. Least absolute shrinkage and selection operator (LASSO) regression was used for feature selection in the training set. Seven supervised models were trained; performance was assessed by area under the receiver-operating characteristic curve (AUC), calibration, and decision-curve analysis. SHAP quantified feature contributions.

**Results:**

LASSO retained 14 features, including 24-hour urine total protein (24UTP), serum creatinine (SCr), age, and atrial dimensions. In the test set, the k-nearest neighbors (KNN) model achieved an AUC of 0.927, with an accuracy of 0.886, sensitivity of 0.920, and specificity of 0.856. Calibration was satisfactory, and decision-curve analysis showed net benefit across commonly used thresholds. Five-fold cross-validation yielded mean AUC 0.90 ± 0.02. SHAP analysis identified proteinuria burden, renal dysfunction, age, and atrial size as major contributors to model output. The final model was translated into a preliminary web-based calculator based on routinely available inputs.

**Conclusions:**

An interpretable ML model incorporating routinely collected clinical and echocardiographic variables showed stable internal performance for identifying comorbid atrial fibrillation in patients with DKD. Because the model is intended to identify concomitant AF status rather than predict incident AF and has undergone internal validation only, further external and prospective validation is required before broader clinical application.

## 1 Introduction

Diabetic kidney disease (DKD), one of the most common microvascular complications of diabetes, is a leading cause of end-stage renal disease and renal replacement therapy (1). DKD not only markedly increases the risk of kidney failure but is also closely associated with thrombosis, stroke, myocardial infarction, and all-cause mortality (2–5). The rising prevalence of DKD poses a major challenge to global health systems (6). Atrial fibrillation (AF), one of the most frequent cardiac arrhythmias, has increased in prevalence by 33% over the past two decades (7). Patients with AF have a 61% higher risk of all-cause death and face greater risks of cardiovascular mortality and stroke (8). Studies have shown that individuals with type 2 diabetes have a higher prevalence of AF than the general population, and this burden further increases among patients with DKD as estimated glomerular filtration rate (eGFR) declines and urinary protein excretion rises (9, 10). DKD and AF share several risk factors, including hypertension and coronary artery disease. Both conditions are influenced by thrombosis, systemic inflammation, and activation of the renin-angiotensin system (RAS), forming a bidirectional relationship (11). On one hand, eGFR decline and proteinuria in DKD may promote atrial remodeling and the presence of AF (12, 13); on the other hand, diabetic patients with AF are more likely to develop DKD, and AF can accelerate renal function deterioration and proteinuria progression in DKD (14, 15). This bidirectional interaction may contribute to worse clinical outcomes.

In clinical practice, AF is often paroxysmal or asymptomatic, particularly in its intermittent forms. Silent AF is frequently underdiagnosed or diagnosed late (16, 17), especially in patients with DKD, who may lack overt arrhythmic symptoms. As a result, opportunities for timely recognition and appropriate rhythm evaluation may be missed. This may contribute to progression of both DKD and AF and increase the risk of serious cardiovascular and cerebrovascular events. These challenges highlight the need for improved recognition of comorbid AF in patients with DKD and for practical, interpretable approaches to support clinical evaluation. Despite the growing clinical burden, no dedicated model for identifying comorbid AF in patients with DKD has been established. Existing tools show limited accuracy in identifying AF among these patients. Previous studies have mainly focused on the general population or on AF assessment outside the specific context of DKD. For example, some investigators combined clinical expertise with artificial intelligence (AI) analysis of 24-hour Holter recordings to improve AF detection rates (18). However, such approaches are difficult to implement widely because DKD patients often lack routine Holter monitoring. Other models based on AI analysis of sinus-rhythm electrocardiograms may aid in identifying paroxysmal AF, but their performance remains limited without incorporation of clinical and laboratory data (19).

Given the increasing prevalence and clinical significance of AF in DKD and the diagnostic limitations of current methods, a reliable and interpretable model to identify comorbid AF status in this population is needed. In this study, we developed and internally validated a machine learning (ML)-based model integrating clinical, laboratory, and echocardiographic variables to identify patients with DKD who have coexisting AF. Our aim was to provide a practical and interpretable tool based on routinely collected inpatient data to support recognition of concomitant AF status in patients with DKD.

## 2 Materials and methods

### 2.1 Study design and setting

This retrospective cohort study included adult patients with DKD who were hospitalized at Dongzhimen Hospital Affiliated to Beijing University of Chinese Medicine, and Beijing Electric Power Hospital between January 2021 and October 2025. A total of 814 hospitalizations were screened, of which 787 unique records met the eligibility criteria and were included in the analysis. The dataset was randomly divided into a training set (70%) and an internal test set (30%) using stratified sampling to preserve the proportion of AF status. Data were extracted by authorized personnel from identifiable electronic medical records to ensure accurate linkage, and all records were de-identified before analysis.

**Figure 1:**
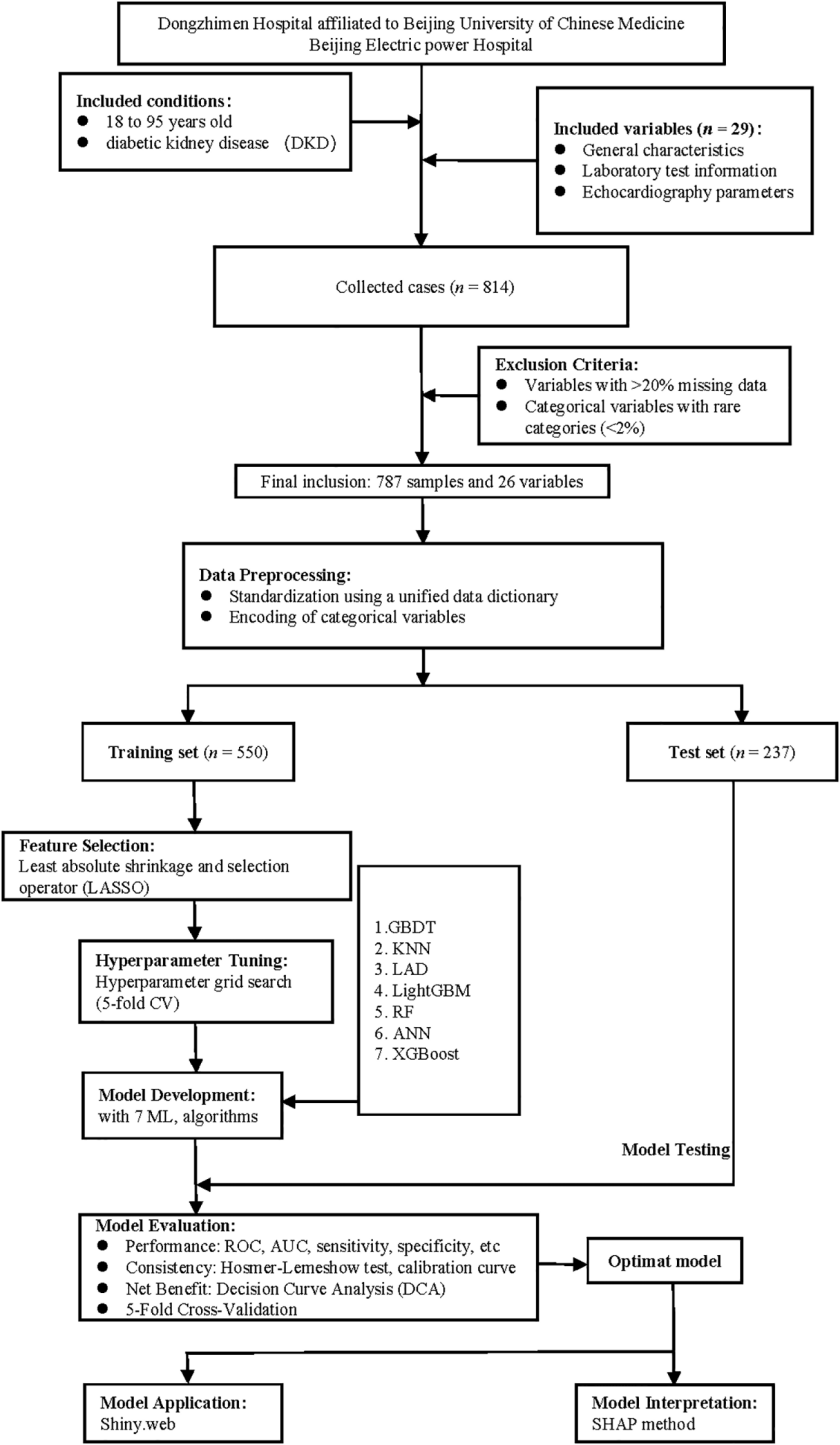
Study workflow and model development framework. The image illustrates the overall process of case selection, data preprocessing, feature selection, model development and evaluation, and result interpretation. A total of 787 eligible records and 26 candidate variables were included and randomly divided into a training set (n = 550) and a test set (n = 237) in a 7:3 ratio.

Ethical approval was obtained from the institutional review board of Dongzhimen Hospital Affiliated to Beijing University of Chinese Medicine (Approval No. 2025DZMEC-607-01). The ethics approval was mutually recognized by the ethics committees of Beijing Electric Power Hospital. Given the retrospective nature of the study, the requirement for informed consent was waived by the institutional review boards. All procedures followed the principles of the Declaration of Helsinki and complied with applicable national regulations. An overview of the study workflow is shown in **Figure 1**.

### 2.2 Endpoint definition

The primary endpoint was documented AF coexisting with DKD. AF was identified using a combination of diagnostic sources, including standard 12-lead electrocardiograms, 24-hour Holter monitoring when available, and International Classification of Diseases, Tenth Revision (ICD-10) diagnostic codes extracted from the electronic medical record. Each case was independently reviewed and confirmed by two physicians to ensure diagnostic accuracy.

Candidate predictors were defined as variables routinely collected during standard inpatient care. For predictors with repeated measurements, the first available value recorded in the medical record was used for analysis. Because a previous diagnosis of AF was not an exclusion criterion, AF may have been present before or documented during the index hospitalization. Accordingly, these predictors were interpreted as routinely collected inpatient features associated with concomitant AF status rather than as measurements guaranteed to precede AF onset. The study objective was to identify concomitant AF status in patients with DKD rather than to predict incident AF.

### 2.3 Participant selection

Participants were identified according to the Chinese Clinical Guidelines for DKD (20) and the diagnostic criteria outlined in the Chinese Guidelines for the Management of AF (2025 edition) (21). Eligible patients were required to meet all of the following inclusion criteria: (1) age between 18 and 95 years (inclusive); (2) both sexes; (3) diagnosis of diabetes mellitus consistent with Chinese and World Health Organization criteria; and (4) diagnosis of diabetic kidney disease confirmed by clinical assessment and laboratory findings in accordance with guideline recommendations.

Exclusion criteria included (1) type 1 diabetes, gestational diabetes, or other specific forms of diabetes; (2) coexisting primary renal diseases such as primary glomerulonephritis, lupus nephritis, or *Henoch-Schönlein purpura nephritis* (3) ongoing systemic glucocorticoid or immunosuppressive therapy; (4) concomitant malignant tumors, decompensated liver cirrhosis, active tuberculosis, infectious shock, disseminated intravascular coagulation, or other critical or terminal illnesses; and (5) major limb amputation within the preceding six months.

Additional cardiac-related exclusions were (1) pacemaker or implantable cardioverter defibrillator implantation; (2) other clinically significant sustained tachyarrhythmias, such as sustained supraventricular tachycardia, excluding atrial flutter; and (3) a history of major cardiac surgery, including valve replacement or coronary artery bypass grafting. A previous diagnosis of atrial fibrillation was not an exclusion criterion because the study focused on identifying comorbid AF status rather than incident or newly diagnosed cases.

### 2.4 Variable collection and measurements

Information extracted from the electronic medical record comprised three domains: (1) demographics and clinical data, such as sex, age, height, weight, blood pressure, and diabetes duration; (2) laboratory tests, including complete blood count, urinalysis, biochemical profiles, arterial blood gas analysis, coagulation parameters, thyroid function, tumor markers, cardiac enzymes, and heart failure biomarkers; and (3) transthoracic echocardiographic findings.

The final predictor set used for model construction consisted of routinely available variables recorded during standard inpatient care, including HbA1c (glycated hemoglobin, %), BUN (blood urea nitrogen, mmol/L), SCr (serum creatinine, μmol/L), BNP (B-type natriuretic peptide, pg/mL), ALT (U/L), AST (U/L), CK-MB (U/L), Myo (myoglobin, ng/mL), WBC (×10^9^/L), RBC (×10¹²/L), 24UTP (24-hour urine total protein, g/24 h), INR, APTT (s), FIB (g/L), LAAPD (left atrial anterior-posterior diameter, mm), LAMLD (left atrial medial–lateral diameter, mm), LASID (left atrial superior-inferior diameter, mm), RAMLD (right atrial medial-lateral diameter, mm), RASID (right atrial superior-inferior diameter, mm), EF (%), and FS (%), as well as sex.

Echocardiography was performed by certified sonographers following a standardized institutional protocol using the same equipment platform. LAAPD was measured in the parasternal long-axis view at end-systole. LAMLD, LASID, and their right-atrial counterparts RAMLD and RASID were obtained from orthogonal apical views in medial-lateral and superior-inferior planes. All diameters were recorded from inner edge to inner edge and averaged over three cardiac cycles. A random 10% subset was re-measured to evaluate intra- and inter-observer consistency, with observed variability below 5%.

These variables were used to characterize the clinical, laboratory, and echocardiographic profile present during routine inpatient assessment and were not intended to represent exposures that necessarily preceded AF onset.

### 2.5 Data preprocessing and handling of missing data

Variables with more than 20% missingness were excluded *a priori*. For the remaining predictors, for which overall missingness was typically less than 10%, missing values were imputed using the MissForest algorithm with default parameter settings and 100 trees. Imputation was performed on the analytic dataset before downstream model development. Categorical variable harmonization followed a unified data dictionary, and rare categories with a prevalence below 2% were merged to ensure model stability.

### 2.6 Feature selection, model development, and hyperparameter tuning

Feature selection was performed using the least absolute shrinkage and selection operator (LASSO) regression in the training dataset. A ten-fold cross-validation procedure was applied using the *glmnet* package in R to identify a parsimonious subset of variables with the strongest discriminative contribution. The optimal penalty parameter (λ) was chosen according to the minimum cross-validated deviance criterion, and the selected features were used for subsequent model construction.

Seven supervised machine learning algorithms were trained using the selected predictors: gradient boosting decision tree (GBDT), k-nearest neighbors (KNN), linear discriminant analysis (LDA), Light Gradient Boosting Machine (LightGBM), random forest (RF), artificial neural network (ANN), and extreme gradient boosting (XGBoost). Each algorithm was trained separately to capture different model structures and learning strategies.

Hyperparameters were tuned using stratified five-fold cross-validation within the training dataset through either grid search or randomized search. For the KNN classifier, hyperparameters were tuned using five-fold GridSearchCV in the training set. Specifically, n_neighbors was searched over the range of 1 to 10, the weighting scheme was evaluated as either uniform or distance, and the distance metric was defined using the Minkowski formulation with p = 1 or p = 2, corresponding to Manhattan and Euclidean distance, respectively. No additional variable standardization or normalization was applied in the implemented workflow. After missing-data handling and variable harmonization, feature selection, model training, and hyperparameter tuning were conducted using the training data only. Because the prevalence of atrial fibrillation was approximately balanced between groups, no oversampling, undersampling, or class weighting was applied. For decision-curve and clinical-impact analyses, probability thresholds were prespecified before evaluation. For classification metrics, binary class labels were generated using each fitted model’s default prediction rule, without additional manual threshold adjustment.

### 2.7 Model evaluation, validation, interpretability, and software

Model performance was evaluated in both the training and test datasets. Discrimination was assessed using the area under the receiver-operating characteristic curve (AUC), with accuracy, sensitivity, specificity, precision, and F1-score as complementary metrics. Calibration was evaluated by the Brier score, calibration intercept and slope, and visual inspection of calibration plots generated with LOESS smoothing. Decision-curve analysis (DCA) was applied to estimate net clinical benefit across prespecified probability thresholds ranging from 5% to 20% for identifying comorbid AF. Clinical-impact curves were used to illustrate the number of individuals classified as positive by the model and the corresponding number of true positives per 1, 000 patients at representative thresholds. Model interpretability was examined for the k-nearest neighbors classifier using KernelSHAP, which quantified the contribution of each predictor to individual model outputs. SHAP values were computed using the same model inputs as those used for KNN training, with a background set of 100 training instances selected by k-medoids sampling to approximate the empirical data distribution.

Descriptive statistical analyses were performed using SPSS version 25.0. Penalized regression for feature selection was conducted in R 4.4.1. All machine-learning models and visualizations, including receiver-operating characteristic, calibration, decision-curve, clinical-impact, and SHAP plots, were implemented in Python 3.10.4 using standard scientific libraries. The final model was further translated into a preliminary web-based calculator. This tool allows users to enter routinely available variables and obtain individualized estimates of comorbid AF likelihood in real time. At this stage, the calculator is intended for preliminary demonstration and internal assessment within the study setting rather than as a substitute for clinical judgment. Because the model has undergone internal validation only, the calculator should not be generalized to other populations or care settings without further external and prospective validation.

## 3 Results

### 3.1 Demographic and baseline characteristics

A total of 787 eligible DKD records were included and randomly divided in a 7:3 ratio into the training set (n = 550) and the test set (n = 237). Overall, 410 patients (52.10%) were in the non-AF group and 377 (47.90%) were in the AF group. In the training set, 287 patients (52.18%) were non-AF and 263 (47.82%) had AF; in the test set, 123 patients (51.90%) were non-AF and 114 (48.10%) had AF. No significant differences were observed between the two sets in age, diabetes duration, HbA1c, SCr, BNP, EF, or sex distribution (male 41.8% vs. 41.5%, P = 0.934), indicating comparable baseline characteristics (**Table 1**).

**Table 1:** Baseline characteristics of the study population across training and test cohorts.

Variables	Total (n = 787)	test (n = 237)	train (n = 550)	*P*
Age, M (Q_1_, Q_3_)	70.00 (59.00, 78.50)	69.00 (59.00, 78.00)	70.00 (59.00, 78.75)	0.624
Duration Of DM, M (Q_1_, Q_3_)	17.00 (10.00, 22.00)	17.36 (10.00, 22.00)	17.00 (10.00, 22.00)	0.699
Height, M (Q_1_, Q_3_)	164.00 (154.95, 174.00)	164.00 (154.90, 173.60)	164.00 (155.00, 174.17)	0.652
Weight, M (Q_1_, Q_3_)	70.00 (62.15, 78.10)	70.00 (62.50, 77.00)	69.78 (62.10, 78.83)	0.845
Hba1c, M (Q_1_, Q_3_)	7.73 (6.62, 8.88)	7.78 (6.65, 8.84)	7.73 (6.59, 8.95)	0.989
BUN, M (Q_1_, Q_3_)	9.20 (6.45, 13.15)	9.60 (6.50, 13.70)	9.05 (6.43, 12.88)	0.366
SCr, M (Q_1_, Q_3_)	109.30 (82.10, 171.30)	107.20 (81.70, 167.60)	110.40 (82.62, 172.45)	0.927
BNP, M (Q_1_, Q_3_)	162.51 (50.08, 315.47)	162.80 (49.59, 308.62)	161.40 (50.26, 325.00)	0.783
AST, M (Q_1_, Q_3_)	19.30 (14.90, 24.45)	19.30 (15.00, 24.00)	19.25 (14.90, 24.67)	0.868
CK-MB, M (Q_1_, Q_3_)	2.80 (1.80, 4.50)	2.70 (1.80, 4.49)	2.80 (1.80, 4.50)	0.739
Myo, M (Q_1_, Q_3_)	56.85 (31.00, 111.43)	55.55 (30.10, 110.90)	57.24 (31.12, 111.51)	0.892
WBC, M (Q_1_, Q_3_)	2.00 (2.00, 6.85)	2.00 (2.00, 6.40)	2.00 (2.00, 7.42)	0.516
RBC, M (Q_1_, Q_3_)	2.00 (2.00, 2.85)	2.00 (2.00, 2.80)	2.00 (2.00, 2.88)	0.988
24UTP, M (Q_1_, Q_3_)	2501.19 (382.10, 5502.69)	1932.20 (313.10, 5495.08)	2567.73 (399.12, 5488.74)	0.478
INR, M (Q_1_, Q_3_)	0.99 (0.90, 1.10)	1.00 (0.91, 1.12)	0.99 (0.90, 1.10)	0.266
APTT, M (Q_1_, Q_3_)	31.80 (28.85, 35.20)	31.80 (28.80, 35.00)	31.80 (28.90, 35.20)	0.622
FIB, M (Q_1_, Q_3_)	3.50 (2.95, 4.20)	3.49 (2.90, 4.20)	3.50 (3.00, 4.20)	0.713
LAAPD, M (Q_1_, Q_3_)	38.20 (33.90, 42.70)	38.10 (34.00, 43.20)	38.25 (33.71, 42.50)	0.697
LAMLD, M (Q_1_, Q_3_)	38.70 (33.02, 44.00)	38.97 (33.06, 44.90)	38.51 (33.01, 43.88)	0.418
LASID, M (Q_1_, Q_3_)	53.00 (46.91, 59.95)	53.00 (46.90, 60.00)	52.95 (46.93, 59.20)	0.475
RAMLD, M (Q_1_, Q_3_)	32.40 (29.00, 37.61)	33.00 (29.30, 38.70)	32.17 (29.00, 37.00)	0.224
RASID, M (Q_1_, Q_3_)	46.10 (41.05, 50.00)	46.00 (41.00, 50.00)	46.20 (41.10, 50.00)	0.878
EF, M (Q_1_, Q_3_)	65.10 (60.31, 69.95)	64.50 (60.60, 69.30)	65.57 (60.20, 70.29)	0.217
FS, M (Q_1_, Q_3_)	36.00 (32.00, 39.20)	35.90 (32.20, 39.00)	36.00 (31.90, 39.38)	0.486
Gender, n(%)				0.934
Male	327 (41.55)	99 (41.77)	228 (41.45)	
Female	460 (58.45)	138 (58.23)	322 (58.55)	

Data are presented as median (Q1, Q3) or n (%). Comparisons between the training and test cohorts used the Wilcoxon rank-sum test for continuous variables and the χ² test (or Fisher’s exact test when expected counts <5) for categorical variables. Two-sided P values; α=0.05.

DM, diabetes mellitus; HbA1c, glycated hemoglobin; BUN, blood urea nitrogen; SCr, serum creatinine; BNP, B-type natriuretic peptide; ALT, alanine aminotransferase; AST, aspartate aminotransferase; CK-MB, creatine kinase-MB; Myo, myoglobin; WBC, white blood cell; RBC, red blood cell; 24UTP, 24-hour urine total protein; INR, international normalized ratio; APTT, activated partial thromboplastin time; FIB, fibrinogen; LAAPD left atrial anterior–posterior diameter; LAMLD left atrial medial-lateral diameter; LASID left atrial superior-inferior diameter; RAMLD right atrial medial-lateral diameter; RASID right atrial superior-inferior diameter; EF ejection fraction; FS fractional shortening; Units and measurement methods are described in Methods.

Within the training set, patients with comorbid AF differed significantly from those without AF (**Table 2**). Patients in the AF group were older (P < 0.001), had a longer duration of diabetes (P = 0.013), and higher body weight (P < 0.001). Although HbA1c levels were slightly lower in the AF group, the difference reached statistical significance (P = 0.023). Renal and cardiac biomarker levels, including BUN, SCr, CK-MB, and myoglobin, were significantly higher in patients with AF (all P < 0.01), and BNP levels were markedly elevated in the AF group (P < 0.001). Echocardiographic assessment showed that atrial dimensions (LAAPD, LAMLD, LASID, RAMLD, and RASID) were significantly larger in the AF group compared with the non-AF group (all P < 0.001), while left ventricular systolic function indices, including EF and FS, were significantly lower in patients with AF (both P < 0.001).

**Table 2:** Baseline characteristics of the study population across training cohorts.

Variables	Total (n = 550)	No Atrial fibrillation (n = 287)	Atrial fibrillation (n = 263)	*P*
Age, M (Q_1_, Q_3_)	70.00 (59.00, 78.75)	62.00 (53.00, 72.00)	75.00 (67.00, 82.00)	**<.001**
Duration Of Dm, M (Q_1_, Q_3_)	17.00 (10.00, 22.00)	16.00 (10.00, 22.00)	18.00 (11.00, 23.00)	**0.013**
Height, M (Q_1_, Q_3_)	164.00 (155.00, 174.17)	163.30 (154.62, 173.86)	165.20 (155.45, 174.85)	0.318
Weight, M (Q_1_, Q_3_)	69.78 (62.10, 78.83)	67.80 (61.55, 75.69)	72.60 (63.35, 80.95)	**<.001**
Hba1c, M (Q_1_, Q_3_)	7.73 (6.59, 8.95)	7.79 (6.71, 9.19)	7.63 (6.54, 8.69)	**0.023**
BUN, M (Q_1_, Q_3_)	9.05 (6.43, 12.88)	8.60 (6.10, 11.45)	9.70 (7.05, 14.85)	**<.001**
SCr, M (Q_1_, Q_3_)	110.40 (82.62, 172.45)	103.70 (78.95, 145.00)	121.30 (88.80, 211.70)	**<.001**
BNP, M (Q_1_, Q_3_)	161.40 (50.26, 325.00)	94.45 (27.58, 182.22)	249.29 (125.24, 445.12)	**<.001**
ALT, M (Q_1_, Q_3_)	14.90 (11.40, 21.55)	13.50 (10.10, 20.65)	15.60 (13.20, 22.10)	**<.001**
AST, M (Q_1_, Q_3_)	19.25 (14.90, 24.67)	18.00 (14.00, 24.30)	20.40 (15.35, 25.10)	**0.022**
CK-MB, M (Q_1_, Q_3_)	2.80 (1.80, 4.50)	2.50 (1.60, 3.90)	3.20 (2.00, 5.63)	**<.001**
Myo, M (Q_1_, Q_3_)	57.24 (31.12, 111.51)	51.00 (28.90, 95.19)	61.00 (33.90, 134.65)	**0.002**
WBC, M (Q_1_, Q_3_)	2.00 (2.00, 7.42)	2.00 (2.00, 9.65)	2.00 (2.00, 4.80)	**0.045**
RBC, M (Q_1_, Q_3_)	2.00 (2.00, 2.88)	2.00 (2.00, 2.90)	2.00 (2.00, 2.75)	0.542
24UTP, M (Q_1_, Q_3_)	2567.73 (399.12, 5488.74)	1800.61 (176.90, 4612.15)	3660.03 (855.95, 6257.25)	**<.001**
INR, M (Q_1_, Q_3_)	0.99 (0.90, 1.10)	1.08 (0.98, 1.21)	0.91 (0.88, 1.00)	**<.001**
APTT, M (Q_1_, Q_3_)	31.80 (28.90, 35.20)	32.20 (28.70, 36.70)	31.57 (29.00, 33.80)	**0.024**
FIB, M (Q_1_, Q_3_)	3.50 (3.00, 4.20)	3.50 (2.90, 4.32)	3.57 (3.00, 4.10)	0.946
LAAPD, M (Q_1_, Q_3_)	38.25 (33.71, 42.50)	35.10 (32.10, 40.20)	40.00 (36.60, 44.30)	**<.001**
LAMLD, M (Q_1_, Q_3_)	38.51 (33.01, 43.88)	35.00 (31.41, 38.85)	42.50 (37.00, 45.95)	**<.001**
LASID, M (Q_1_, Q_3_)	52.95 (46.93, 59.20)	49.02 (45.17, 53.58)	57.50 (50.35, 60.00)	**<.001**
RAMLD, M (Q_1_, Q_3_)	32.17 (29.00, 37.00)	30.20 (27.85, 32.90)	35.40 (31.00, 41.20)	**<.001**
RASID, M (Q_1_, Q_3_)	46.20 (41.10, 50.00)	43.80 (40.75, 47.30)	49.80 (42.45, 50.00)	**<.001**
EF, M (Q_1_, Q_3_)	65.57 (60.20, 70.29)	66.76 (63.45, 71.10)	63.30 (57.00, 69.45)	**<.001**
FS, M (Q_1_, Q_3_)	36.00 (31.90, 39.38)	36.90 (34.33, 40.00)	34.40 (29.10, 38.75)	**<.001**
Gender, n(%)				**<.001**
Male	228 (41.45)	147 (51.22)	81 (30.80)	
Female	322 (58.55)	140 (48.78)	182 (69.20)	

Data are presented as median (Q1, Q3) or n (%). Group comparisons (No AF vs AF) used the Wilcoxon rank-sum test for continuous variables and the χ² test or Fisher’s exact test for categorical variables (when expected counts <5). Two-sided P values; α=0.05. AF indicates atrial fibrillation (clinically diagnosed). Other abbreviations as in **Table 1**.

DM, diabetes mellitus; HbA1c, glycated hemoglobin; BUN, blood urea nitrogen; SCr, serum creatinine; BNP, B-type natriuretic peptide; ALT, alanine aminotransferase; AST, aspartate aminotransferase; CK-MB, creatine kinase-MB; Myo, myoglobin; WBC, white blood cell; RBC, red blood cell; 24UTP, 24-hour urine total protein; INR, international normalized ratio; APTT, activated partial thromboplastin time; FIB, fibrinogen; LAAPD left atrial anterior–posterior diameter; LAMLD left atrial medial-lateral diameter; LASID left atrial superior-inferior diameter; RAMLD right atrial medial-lateral diameter; RASID right atrial superior-inferior diameter; EF ejection fraction; FS fractional shortening; Units and measurement methods are described in Methods.

Bold values indicate elements with statistical significance (p<0.05).

In addition, the proportion of women was significantly higher in the AF group than in the non-AF group (69.2% vs. 48.8%, P < 0.001).

### 3.2 Feature selection

Feature selection was performed using the LASSO regression with tenfold cross-validation in the training set. This approach retained variables with the strongest discriminative contribution. Model performance metrics were monitored across iterations to identify the subset with the best cross-validated performance.

**Figure 2:**
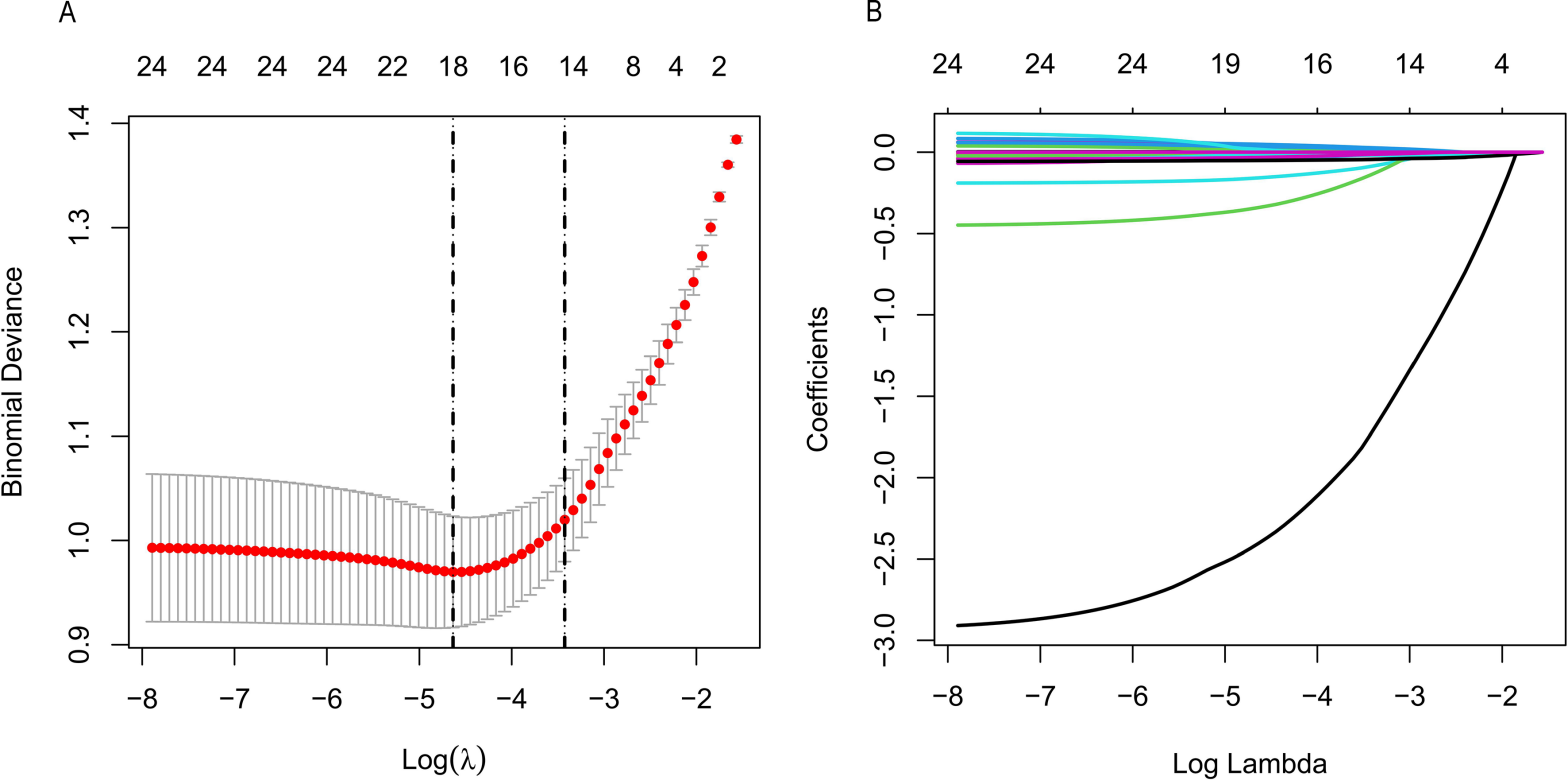
LASSO feature selection. **(A)** The curve showing the change in binomial deviance with log(λ) under 10-fold cross-validation, with vertical dashed lines indicating λ_min and λ_1se, and the top axis showing the number of non-zero coefficients at different penalty strengths. **(B)** Coefficient path plot: The regression coefficients of each candidate variable gradually shrink toward zero as log(λ) increases, with the final non-zero coefficients forming the selected feature set for the model.

As shown in **Figure 2A**, the mean cross-validation curve indicated stable convergence, and the corresponding coefficient profiles are presented in **Figure 2B**. Fourteen variables with non-zero coefficients were ultimately retained: 24UTP, SCr, age, LAMLD, weight, LASID, LAAPD, RAMLD, FS, BUN, HbA1c, CK-MB, FIB, and INR. These features were subsequently used for model development.

### 3.3 Model performance comparison

**Figure 3:**
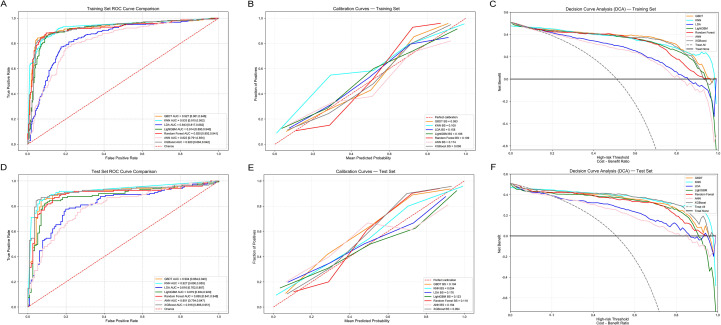
Comparison of model performance across multiple algorithms in the training and test sets. **(A)** Receiver operating characteristic (ROC) curves in the training set for the seven candidate algorithms; the dashed diagonal line indicates chance-level discrimination. **(B)** Calibration curves in the training set. The diagonal dashed line represents perfect agreement between predicted and observed probabilities. The Brier score, shown in the panel, quantifies the overall error of probabilistic predictions, with lower values indicating better calibration and predictive accuracy. **(C)** Decision-curve analysis (DCA) in the training set. Net benefit is plotted across a range of threshold probabilities, with the “treat-all” and “treat-none” strategies shown as reference lines. **(D)** ROC curves in the test set. **(E)** Calibration curves in the test set, with the Brier score annotated; lower values indicate better agreement between predicted risk and observed outcome. **(F)** DCA in the test set.

Seven supervised machine-learning algorithms were trained using the selected features: KNN, RF, XGBoost, LightGBM, GBDT, LDA, and ANN. In the training set, all models showed acceptable to good discrimination (**Figure 3A**). The KNN model achieved the highest AUC (0.933) with an accuracy of 0.8745, sensitivity of 0.9077, specificity of 0.8448, and a Brier score of 0.100. RF, XGBoost, and LightGBM performed comparably, with AUCs of 0.9200, 0.9196, and 0.9144, respectively (**Table 3**).

**Table 3:** Performance of seven machine learning models in the training and test sets.

Data set	Model	Accuracy	Sensitivity	Specificity	F1	AUC	Brier
Train	GBDT	0.8873	0.9195	0.8581	0.8856	0.9266	0.093
	KNN	0.8745	0.9077	0.8448	0.8725	0.9326	0.100
	LDA	0.7891	0.8022	0.7762	0.7906	0.8427	0.158
	LightGBM	0.8800	0.8967	0.8638	0.8804	0.9144	0.106
	RF	0.8855	0.9098	0.8627	0.8848	0.9200	0.109
	ANN	0.7673	0.7508	0.7878	0.7816	0.8220	0.098
	XGBoost	0.8818	0.9060	0.8592	0.8812	0.9196	0.094
Test	GBDT	0.8819	0.9266	0.8438	0.8783	0.9042	0.104
	KNN	0.8861	0.9196	0.8560	0.8841	0.9271	0.094
	LDA	0.7806	0.8000	0.7623	0.7797	0.8164	0.176
	LightGBM	0.8650	0.9009	0.8333	0.8621	0.8791	0.123
	RF	0.8523	0.8981	0.8140	0.8472	0.8955	0.118
	ANN	0.7468	0.7521	0.7414	0.7521	0.8012	0.194
	XGBoost	0.8945	0.9444	0.8527	0.8908	0.9162	0.094

Performance is reported on the training and held-out test sets. Accuracy, Sensitivity (recall), Specificity, F1-score, AUC (area under the ROC curve), and Brier score (lower is better) are shown.

Models: GBDT, gradient boosting decision tree; KNN, k-nearest neighbors; LDA, linear discriminant analysis; LightGBM, Light Gradient Boosting Machine; RF, random forest; ANN, artificial neural network; XGBoost, extreme gradient boosting.

In the test set (**Figure 3D**), the KNN model maintained the best overall performance, with an AUC of 0.9271, an accuracy of 0.8861, a sensitivity of 0.9196, and a specificity of 0.8560. XGBoost achieved the lowest Brier score (0.094), while RF and LightGBM yielded AUCs of 0.8955 and 0.8791, respectively. Although LightGBM had a slightly higher Brier score (0.123), its calibration remained within an acceptable range (**Table 3**).

Calibration curves showed good agreement between model-estimated and observed probabilities for all models (**Figures 3B, E**). Decision-curve analysis indicated that KNN and XGBoost provided the greatest net clinical benefit across most threshold ranges for identifying comorbid AF (**Figures 3C, F**). Five-fold cross-validation further confirmed the robustness of model performance, with an average AUC of 0.90 ± 0.02 (**Supplementary Figure 1**). Taken together, KNN demonstrated the most favorable balance of discrimination, calibration, and generalization among the evaluated models.

In sensitivity analyses excluding INR and FIB, the KNN modeling procedure was repeated under three different stratified 7:3 random splits (random seeds 42, 123, and 2024). The resulting test-set AUCs were 0.774, 0.800, and 0.819, respectively. Accuracy, sensitivity, specificity, precision, F1-score, and Brier score also remained within a relatively narrow range across the three splits. These findings indicate that model performance decreased after exclusion of INR and FIB but remained acceptable across the three data splits (**Supplementary Table 2**).

### 3.4 Model interpretation and SHAP analysis

**Figure 4:**
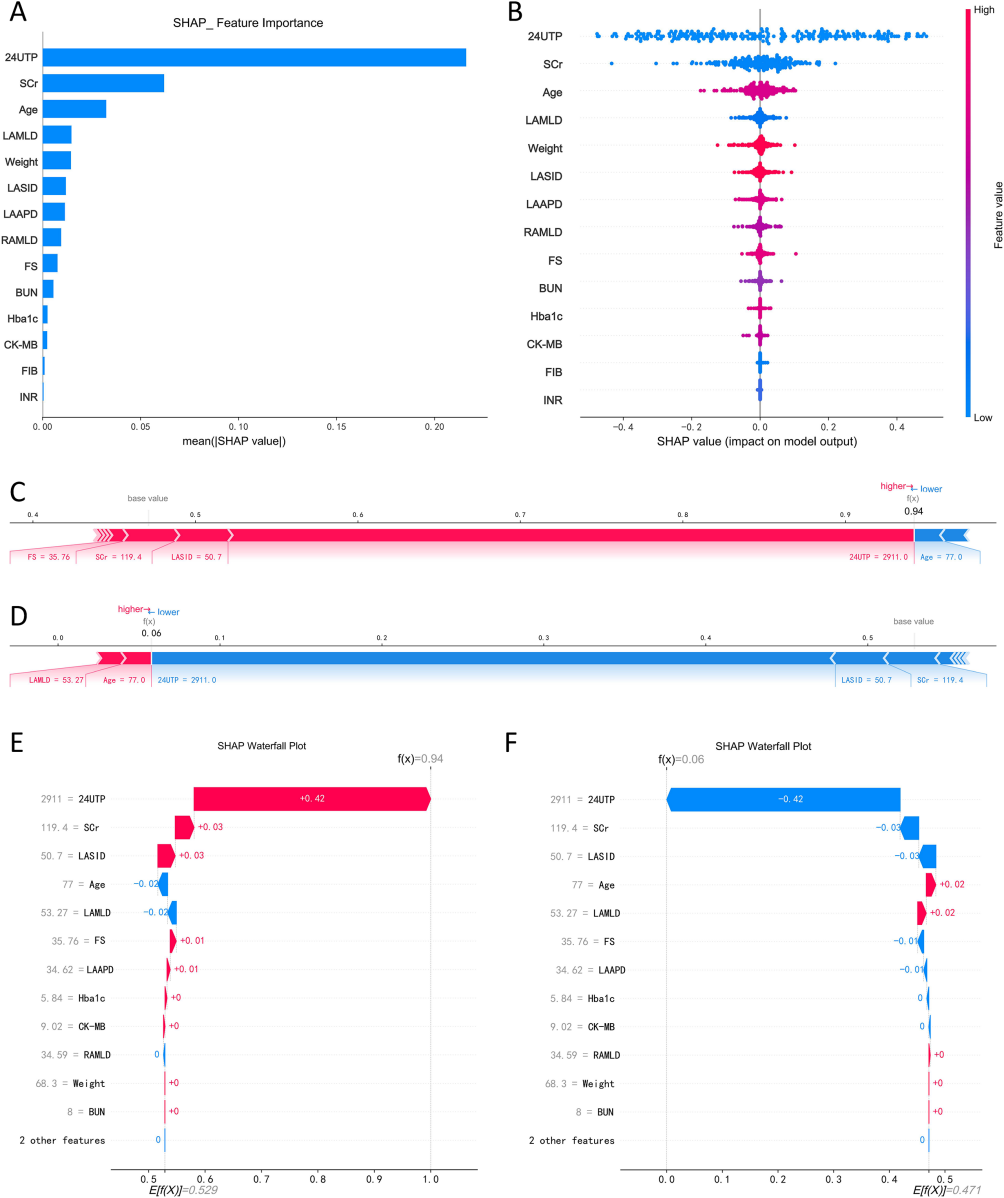
SHAP interpretability analysis of the optimal KNN model. **(A)** SHAP feature importance plot showing the overall contribution of variables to model output, with 24UTP, SCr, and age-related indicators ranking among the most influential features. **(B)** SHAP summary plot. Each point represents one individual. The horizontal position indicates the direction and magnitude of a feature’s contribution to the model output: positive SHAP values push the prediction toward atrial fibrillation, whereas negative SHAP values push it away. Color represents the relative feature value (high or low). **(C, D)** Decision plot and force plot for representative individuals, illustrating how multiple features cumulatively increase or decrease the model-predicted probability. Red and blue indicate positive and negative contributions, respectively, and the endpoint represents the final estimated probability. **(E, F)** Waterfall plots showing the stepwise contribution of each feature to the final model prediction for representative cases. Notably, SHAP analysis reflects data-driven contributions within the trained model and should be interpreted as model explanation rather than evidence of causality. For certain echocardiographic variables, including atrial dimensions, the direction of association in this cohort differs from conventional structural paradigms of atrial fibrillation, highlighting the nonlinear and context-specific nature of model-based prediction.

To explore the interpretability of the KNN model and quantify the contribution of each feature, SHAP analysis was applied (**Figure 4**). The mean absolute SHAP value plot (**Figure 4A**) identified age, SCr, LAAPD, and 24UTP as the features with the greatest contribution to model output. Among these features, 24UTP showed the highest mean SHAP value, indicating the greatest overall contribution to model output.

The SHAP summary plot (**Figure 4B**) illustrated the distribution of feature effects across all samples. Each point represents a single observation, with color indicating the feature value (red for high, blue for low) and position reflecting its positive or negative contribution to the model output. SHAP contributions varied across the feature value range, with both positive and negative contributions observed depending on the variable and its magnitude.

To visualize individual-level model outputs, representative force and waterfall plots were generated (**Figures 4C–F**). These plots demonstrated how individual features shifted the model output toward or away from the AF classification, highlighting the cumulative contribution of multiple features. Overall, proteinuria burden and renal function measures were among the major contributors to model output.

### 3.5 Clinical impact curve analysis

**Figure 5:**
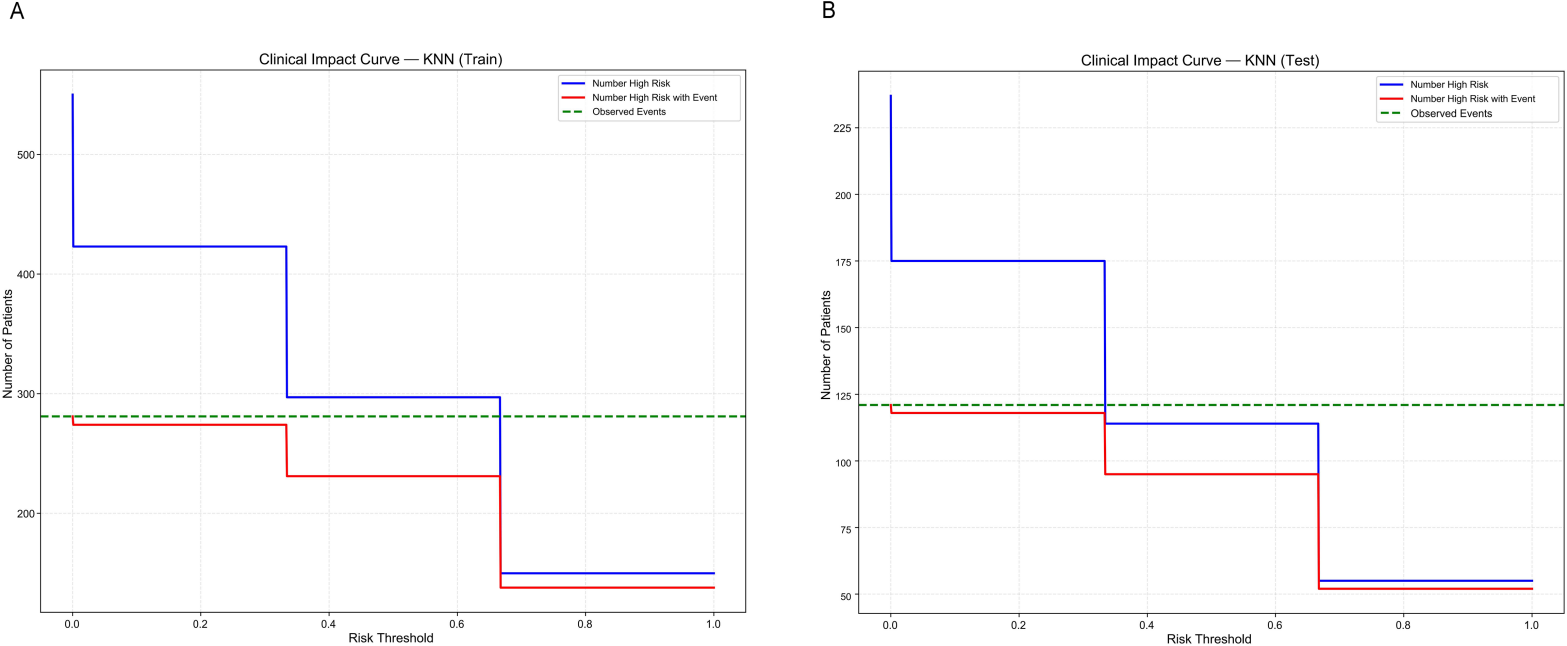
Clinical impact curve analysis (CIC) of the optimal KNN model. Clinical impact curves for the training set **(A)** and test set **(B)** across different threshold probabilities. The blue line represents the number of individuals classified by the model as high risk (predicted positive) at each threshold. The red line represents the number of true-positive cases among those identified as high risk. The green dashed line represents the observed number of atrial fibrillation cases in the dataset. Together, these curves illustrate the clinical implications of using different decision thresholds.

To assess the clinical utility of the model, clinical impact curves (CICs) for the KNN classifier were generated in both the training and test sets (**Figures 5A, B**). As the decision threshold increased, the number of patients classified as positive by the model (blue line) gradually decreased. The number of true positives (red line) closely overlapped with the number of observed AF cases (green dashed line), indicating good agreement between model classifications and observed AF status.

Across commonly used decision thresholds, the KNN model consistently demonstrated favorable clinical utility and net benefit. The training and test set curves showed similar patterns, consistent with stable internal performance.

### 3.6 Web calculator application

**Figure 6:**
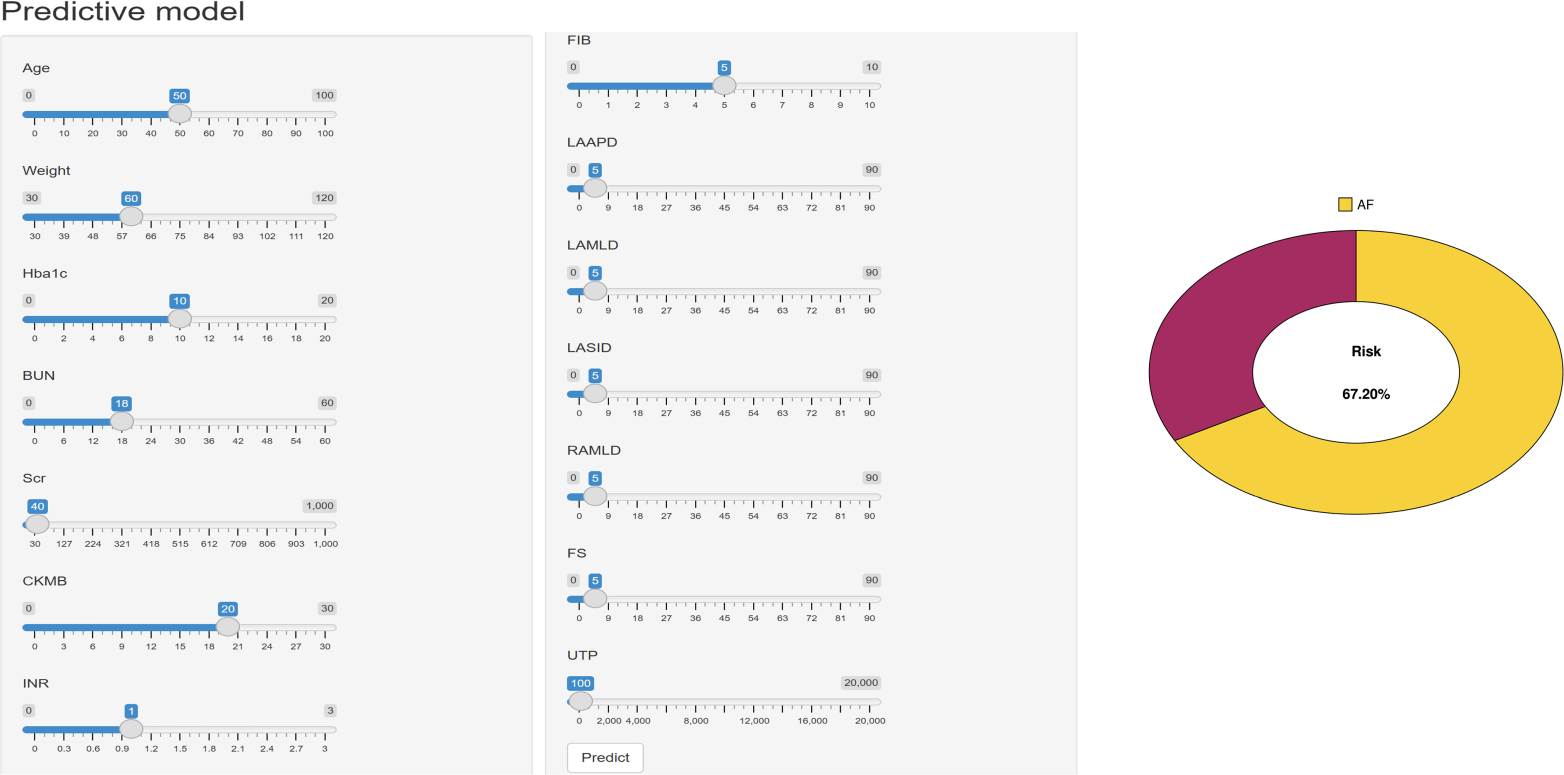
User interface of the web-based calculator derived from the final KNN model. The image shows the web-based calculator developed from the final KNN model using the Shiny framework. Users can input 14 routinely available variables to obtain individualized estimates of comorbid atrial fibrillation likelihood in patients with diabetic kidney disease. The calculator is presented here as a preliminary prototype derived from the internally validated model.

The final KNN model was translated into a preliminary web-based calculator for use within the study setting (**Figure 6**). The interface allows users to enter 14 routinely available variables and obtain an individualized estimate of comorbid AF likelihood in real time. The output provides the model-estimated probability together with a categorical classification label to facilitate interpretation. The prototype is currently available through a web-based interface for demonstration purposes: https://endocrine-dzm-af-prediction-model.shinyapps.io/shiny/.

## 4 Discussion

To our knowledge, this is the first machine learning-based clinical model developed to identify comorbid AF in patients with DKD. Using routinely collected clinical, laboratory, and echocardiographic data, we compared seven ML algorithms. In our dataset, KNN showed the best overall performance. KNN is a nonparametric supervised method that classifies a sample according to its nearest neighbors, requires relatively few hyperparameters, and is straightforward to implement (22). Prior studies have used KNN for clinical risk stratification in sleep apnea, coronary artery disease, and AF among intensive care unit patients (23, 24). To improve transparency, we applied SHAP to interpret model outputs and quantify feature contributions, consistent with earlier AF modeling studies (25, 26). Key features highlighted by SHAP were clinically plausible and readily obtainable during routine inpatient evaluation. We also implemented a web calculator as a preliminary demonstration tool based on the internally validated model. Because the required inputs are routinely available in inpatient practice, this prototype may support internal assessment within similar study settings, although broader applicability requires independent external validation. Because patients with pre-existing AF were not excluded and predictors were derived from routinely collected inpatient measurements, some variables may reflect correlates or consequences of established AF. The model should therefore be interpreted primarily as identifying DKD patients with concomitant AF-associated clinical phenotypes rather than predicting future AF onset. In clinical practice, the model may be used as a supplementary tool in hospitalized patients with DKD. It may help identify those who need closer rhythm surveillance or further ECG or Holter evaluation.

The selected features mainly reflected two domains: renal metabolic burden and cardiac structural and functional remodeling. Together, these patterns were biologically relevant to comorbid AF in DKD. Indicators of renal impairment and proteinuria emerged as dominant signals. Higher SCr and greater 24UTP were consistent with prior studies showing that kidney dysfunction and protein loss are associated with AF occurrence, often in a dose-response pattern (12, 13, 27). BUN showed similar associations and has also been retained in critical care AF modeling studies (25, 28). From a pathophysiological perspective, these findings support the concept of cardiorenal interaction in DKD. Impaired renal function, persistent proteinuria, systemic inflammation, activation of the renin-angiotensin-aldosterone system, and sodium-water retention may jointly contribute to volume overload, atrial stretch, and progressive atrial remodeling, all of which are relevant to AF-associated clinical phenotypes (29). In parallel, aging and obesity were strong clinical correlates. The risk of AF increases steadily with advancing age, likely because of atrial remodeling, reduced conduction velocity, and sinoatrial node dysfunction (30–33). Excess body weight is also associated with incident AF and with progression from paroxysmal to persistent AF through mechanisms such as hemodynamic overload, hypertension, inflammation, and oxidative stress (34–37). Obesity is also strongly linked to the onset and progression of DKD, highlighting a shared metabolic and structural risk pathway (38–40).

Cardiac structural and functional variables further complemented these metabolic indicators. Enlarged atrial dimensions on echocardiography correlated with declining eGFR and adverse cardiovascular profiles among DKD patients (41–44). Left atrial volume index was independently associated with new-onset AF and adverse outcomes regardless of ejection fraction or chamber geometry, consistent with chronic volume load, inflammation, and neurohormonal activation (45–48). For the right atrium, genetic evidence from Mendelian randomization has suggested a link between reduced eGFR and atrial enlargement, although DKD-specific clinical studies remain limited (49). Reduced systolic performance, reflected by fractional shortening, was also consistent with the interlinked pathophysiology of AF, heart failure, and DKD (50–54). Importantly, model-derived associations do not necessarily mirror conventional structural paradigms on a one-to-one basis. SHAP reflects the contribution of each variable within a multivariable, nonlinear prediction framework rather than an isolated causal effect. Accordingly, the direction or relative importance of certain echocardiographic features may differ from expectations derived from single-parameter or univariable analyses. Other variables, including HbA1c, CK-MB, fibrinogen, and INR, may provide additional clinical context by reflecting glycemic exposure, myocardial injury, coagulation activity, and anticoagulation status (8, 55–61). In addition, sensitivity analyses excluding INR and FIB showed that KNN performance decreased but remained acceptable across different stratified 7:3 random splits, suggesting that the main findings were not solely driven by these two coagulation-related variables. However, these coagulation-related variables should be interpreted cautiously, as they may partly reflect treatment-related factors, particularly anticoagulation exposure, rather than underlying disease biology alone.

Overall, we developed an interpretable ML-based model to identify comorbid AF in patients with DKD using routinely available clinical, laboratory, and echocardiographic data. In our dataset, the model showed good discrimination and calibration, and we implemented a web calculator as a preliminary prototype based on the internally validated model. However, this study also has the following limitations. First, the retrospective design introduces risks of selection and information bias, and reliance on existing records may lead to missing or misclassified data. Second, although the dataset was derived from two hospitals, all participants were recruited in China. Both the model and the accompanying web calculator were internally validated only, and their performance in other ethnic, geographic, and clinical settings remains uncertain. In addition, AF prevalence was relatively balanced in both the training and test sets. Although this may have facilitated model development and internal validation, AF prevalence may differ substantially across real-world populations and care settings. Because predictive values depend on disease prevalence, the PPV of the model may decrease and the NPV may increase in lower-prevalence settings. Therefore, the clinical interpretation and transportability of model performance may vary, underscoring the need for external validation in cohorts with different case mixes. Third, the model included coagulation indices such as FIB and INR, and we did not exclude patients receiving anticoagulants; although these features contributed modestly, we cannot determine whether anticoagulation affected calibration. Fourth, the sample size was moderate, which may limit statistical power and the precision of subgroup effects. Future studies should include larger cohorts, additional centers, and external as well as prospective validation.

## 5 Conclusions

This study developed and internally validated an interpretable machine-learning model to identify comorbid atrial fibrillation among patients with diabetic kidney disease using routinely collected inpatient clinical, laboratory, and echocardiographic variables. The model showed good discrimination and calibration within the study cohort, and SHAP analysis improved interpretability by highlighting cardiorenal and atrial structural features relevant to AF classification. A web-based calculator was implemented as a preliminary prototype to support internal assessment of the model. However, because the model is intended to identify concomitant AF rather than predict incident AF, and because both the model and calculator have undergone internal validation only, further external and prospective validation in independent cohorts is required before broader clinical application.

## Data Availability

The raw data supporting the conclusions of this article will be made available by the authors, without undue reservation.

## Supplementary material

Supplementary Figure 1 ROC curve and average AUC under five-fold cross-validation. ROC curves from five-fold cross-validation of the final model. Lighter-colored curves represent the individual folds, and the dark blue curve represents the average ROC curve. The mean AUC is approximately 0.90 ± 0.02, indicating consistent discriminatory performance during internal cross-validation.
