# Supplementary figures and images for: Development and internal validation of an interpretable machine-learning model for identifying comorbid atrial fibrillation in patients with diabetic kidney disease
Source: Front Clin Diabetes Healthc. 2026 May 26;7:1785125. doi: 10.3389/fcdhc.2026.1785125 (PMC13246348; doi:10.3389/fcdhc.2026.1785125)

5-Fold Cross-Validation ROC Curve

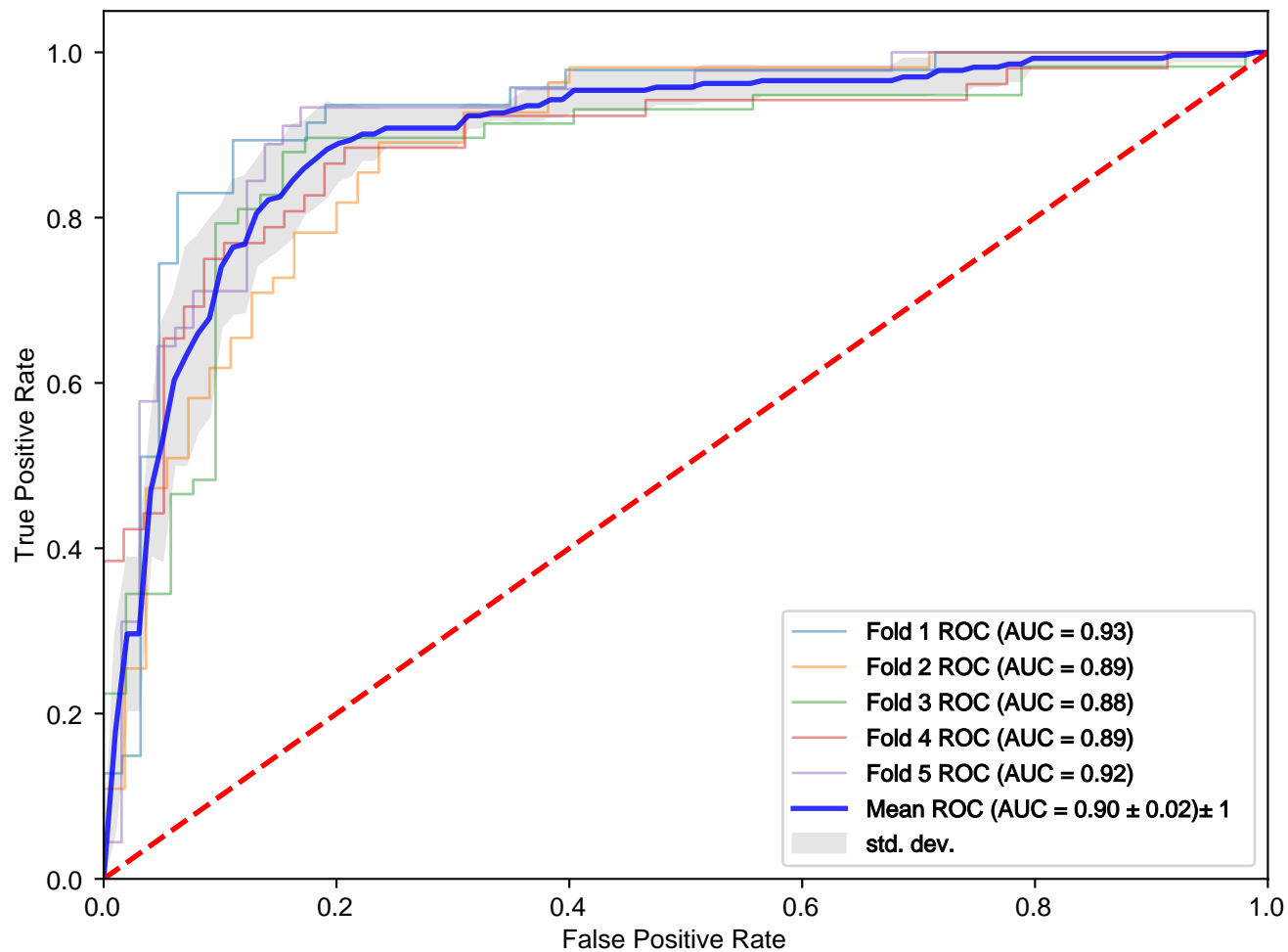

Supplement: Supplementary Figure 1 — ROC curve and average AUC under five-fold cross-validation. ROC curves from five-fold cross-validation of the final model. Lighter-colored curves represent the individual folds, and the dark blue curve represents the average ROC curve. The mean AUC is approximately 0.90 ± 0.02, indicating consistent discriminatory performance during internal cross-validation. [file Image1.pdf]
