# Supplementary material for: Development and internal validation of an interpretable machine-learning model for identifying comorbid atrial fibrillation in patients with diabetic kidney disease
Source: Front Clin Diabetes Healthc. 2026 May 26;7:1785125. doi: 10.3389/fcdhc.2026.1785125 (PMC13246348; doi:10.3389/fcdhc.2026.1785125)
Supplement: Supplementary file 2 [file Table1.docx]

**Table S1. Variable-wise missingness and handling strategy before model development.**

| **Variable** | **Missing, n** | **Missingness, %** | **Handling strategy** |
| --- | --- | --- | --- |
| Height | 16 | 2.0 | Imputed using MissForest |
| Weight | 16 | 2.0 | Imputed using MissForest |
| BUN | 31 | 4.0 | Imputed using MissForest |
| BNP | 31 | 4.0 | Imputed using MissForest |
| CK-MB | 47 | 6.0 | Imputed using MissForest |
| 24UTP | 67 | 8.5 | Imputed using MissForest |
| TSH | 181 | 23.0 | Excluded before modeling (>20% missingness) |
| FT3 | 181 | 23.0 | Excluded before modeling (>20% missingness) |
| FT4 | 181 | 23.0 | Excluded before modeling (>20% missingness) |

**Notes:** Missingness was assessed in the full analytic cohort (n = 787). Only variables with non-zero missingness are shown. Variables with missingness >20% were excluded before model development. For the remaining variables, missing values were imputed using the MissForest algorithm before downstream model development.

**Abbreviations:** BUN, blood urea nitrogen; BNP, B-type natriuretic peptide; CK-MB, creatine kinase-MB; 24UTP, 24-hour urine total protein; TSH, thyroid-stimulating hormone; FT3, free triiodothyronine; FT4, free thyroxine.
