# Supplementary material for: Development and internal validation of an interpretable machine-learning model for identifying comorbid atrial fibrillation in patients with diabetic kidney disease
Source: Front Clin Diabetes Healthc. 2026 May 26;7:1785125. doi: 10.3389/fcdhc.2026.1785125 (PMC13246348; doi:10.3389/fcdhc.2026.1785125)
Supplement: Supplementary file 3 [file Table2.docx]

Table S2. Sensitivity analysis of the KNN model after exclusion of INR and FIB under different stratified 7:3 random splits.

| Random  seed | n_neighbors | Weights | p | 5-fold CV AUC | Test AUC | Accuracy | Sensitivity | Specificity | Precision | F1-score | Brier score |
| --- | --- | --- | --- | --- | --- | --- | --- | --- | --- | --- | --- |
| 42 | 3 | distance | 1 | 0.821 ± 0.033 | 0.774 | 0.730 | 0.763 | 0.699 | 0.702 | 0.731 | 0.212 |
| 123 | 4 | distance | 1 | 0.820 ± 0.034 | 0.800 | 0.738 | 0.746 | 0.732 | 0.720 | 0.733 | 0.189 |
| 2024 | 7 | distance | 1 | 0.812 ± 0.015 | 0.819 | 0.755 | 0.798 | 0.715 | 0.722 | 0.758 | 0.176 |

Abbreviations: KNN, k-nearest neighbors; INR, international normalized ratio; FIB, fibrinogen; CV, cross-validation; AUC, area under the receiver operating characteristic curve.

Notes: Sensitivity analyses were performed by excluding INR and FIB and repeating KNN modeling under three different stratified 7:3 random splits (random seeds 42, 123, and 2024). Hyperparameters were tuned by five-fold cross-validation within the training set.
